# Social capital interventions for human papillomavirus (HPV) immunization and cervical cancer screening: A rapid review

**DOI:** 10.14745/ccdr.v50i78a04

**Published:** 2024-07-24

**Authors:** Christina Gillies, Lisa K Allen-Scott, Candace I J Nykiforuk, Ana Paula Belon, Minji Olivia Kim, Bernice Lee, Laura Nieuwendyk, Kamala Adhikari, Elaine M Ori

**Affiliations:** 1 Provincial Population and Public Health, Alberta Health Services, Edmonton, AB; 2 School of Public Health, University of Alberta, Edmonton, AB; 3 Centre for Healthy Communities, School of Public Health, University of Alberta, Edmonton, AB; 4 Department of Community Health Sciences, University of Calgary, Calgary, AB; 5 Department of Oncology, University of Calgary, Calgary, AB; 6 Department of Health, Community & Education, Mount Royal University, Calgary, AB

**Keywords:** cervical cancer, HPV vaccination, cancer screening, social capital, social support, health equity, public health

## Abstract

**Background:**

Social capital can be used as a conceptual framework to include social context as a predictor of human papillomavirus (HPV) vaccination and cervical cancer screening behaviours. However, the effectiveness of interventions that use social capital as a mechanism to improve uptake of immunization and screening remains elusive.

**Objective:**

To synthesize empirical evidence on the impact of social capital interventions on HPV immunization and cervical cancer screening and describe key characteristics of such interventions.

**Methods:**

Using a rapid review methodology, a search of literature published between 2012 and 2022 was conducted in four databases. Two researchers assessed the studies according to inclusion criteria in a three-step screening process. Studies were assessed for quality and data concerning social capital and equity components and intervention impact were extracted and analyzed using narrative synthesis.

**Results:**

Seven studies met the inclusion criteria. Studies found improved knowledge, beliefs and intentions regarding HPV immunization and cervical cancer screening. None of the studies improved uptake of immunization; however, three studies found post-intervention improvements in uptake of cervical cancer screening. All studies either tailored their interventions to meet the needs of specific groups or described results for specific disadvantaged groups.

**Conclusion:**

Limited evidence suggests that interventions that consider and reflect local context through social capital may be more likely to increase the uptake of HPV immunization and cervical cancer screening. However, further research must be done to bridge the gap in translating improvements in knowledge and intention into HPV immunization and cervical cancer screening behaviours.

## Introduction

Human papillomavirus (HPV) is the most common sexually transmitted infection in North America, affecting most sexually active people at least once in their lifetime, if not immunized ((1)). Persistent HPV infection can cause cancers of the cervix, as well as the vulva, vagina, penis, anus, mouth and throat ((2,3)). While cervical cancer incidence has slowly declined, it remains the third most common cancer among people with a cervix aged 35–44 years ((4)). Due to social and structural determinants, inequities in HPV infection rates and incidence of cervical cancer are also experienced by Indigenous people, immigrants, sexual and gender minorities and residents in rural and remote communities ((1,5)). Therefore, slowing the spread of HPV infection and eliminating the incidence of cervical cancer through evidence-based, equitable interventions to improve prevention remains a pressing public health concern.

Morbidity and mortality of cervical cancer can be reduced or eliminated through primary and secondary prevention against HPV. In Canada, publicly funded vaccination programs in school, community and healthcare settings ((6)) have proven to be a highly effective primary prevention strategy for HPV infection and high-risk precancerous cervical lesions ((1)). Secondary prevention through publicly funded cervical cancer screening programs (e.g., Pap smears and self-sampling test kits) can also detect cell changes to be treated before they progress to cervical cancer ((4)). The provincial and territorial final dose uptake rate for HPV vaccination in schools ranges from 57% to 91% ((7)), while adherence to recommended cervical cancer screening guidelines across the country also ranges, from 63% to 71% ((4)).

Human papillomavirus immunization and cervical cancer screening behaviours are complex and influenced by numerous factors, including lack of information, vaccine hesitancy and gaps in access and financial coverage (6,8). Social capital has been used as a conceptual framework to broaden the lens beyond conventional predictors of immunization and screening behaviours to include social context. Within public health, social capital most often refers to the resources available to people through their social networks (e.g., families, workplaces) ((9)). Indicators of social capital fall into two dimensions: cognitive social capital (subjective perception of level of trust, sharing and reciprocity) and structural social capital (observable extent of social participation) ((9)). Social capital is further understood through three functions: bonding social capital (resources accessed within groups that have similar socioeconomic and demographic characteristics), bridging social capital (resources that may be accessed across groups with different characteristics) and linking social capital (networks of trust connecting groups with differences in power) ((9)).

Social capital interventions represent activities aimed at improving health through changes in an individual’s or group’s capacity to mobilize social capital ((9)), including social norms, social cohesion, community networks, connectedness, belonging and reciprocity. For instance, social capital may help provide underserved individuals with information, financial assistance or transportation to access immunization programs. Such interventions may enhance individual uptake of cancer prevention behaviours, thereby reducing cancer incidence and mitigating cancer-related inequities ((8)). However, there is limited knowledge concerning social capital as a mechanism to improve uptake of HPV immunization and cervical cancer screening. This paper aimed to synthesize empirical evidence on the impact of social capital interventions on HPV immunization and cervical cancer screening and describe key characteristics of such interventions.

## Methods

Evidence concerning social capital and HPV-related cancer prevention was required for the development of a provincial-based intervention to reduce HPV-related cancers in Alberta. Accordingly, a rapid review methodology ((10,11)) was chosen for evidence-based, rapid decision-making. The research question was: What is the empirical evidence of the impact of social capital interventions on uptake of HPV immunization and/or cervical cancer screening (secondary prevention) to prevent HPV-associated cancers?

The search strategy was developed by a librarian in collaboration with content experts, from May 6 to June 22, 2022. The search strategy included testing, language, development, peer review, translations and deduping. The search was conducted in Ovid Medline, Ovid PsycINFO, Ovid Embase and EBSCOhost CINAHL on June 22, 2022 (the search protocol, including full search strategies, is available upon request). Studies were included if they were peer-reviewed intervention studies, systematic reviews, or meta-analyses published in English between 2012 and 2022 (see **Appendix**, **Table A1** for inclusion and exclusion criteria).

Following a three-step screening process, two researchers began by independently conducting title-abstract screening for the same set of 10% of the studies. A third researcher helped resolve discrepancies. When an inter-rater agreement of 100% was reached, the database was split into two. The same two researchers completed the primary screening separately using half of the database each. This process was repeated for full text screening. Finally, the references of included studies were screened for potential inclusion. No protocol outlining all methodological steps in our rapid review was developed *a priori* or registered in an open-source platform.

One researcher extracted data (e.g., participants’ characteristics, study limitations) from the studies using Microsoft Excel and a second researcher verified the data (available upon request). Through group discussion, social capital was categorized by dimensions and functions. The PROGRESS-Plus ((12)) characteristics from Cochrane Equity were used to organize findings by social factors influencing health inequities. Quality appraisal was performed independently by two researchers for 10% of studies using the Quality Assessment Tool for Quantitative Studies (13). After achieving an inter-rater agreement of 100%, the two researchers completed the remaining quality appraisals. They discussed their independent scoring with each other to determine the final rating (see Appendix, **Table A2**). The 2020 PRISMA checklist (14) was used as a reporting guideline for our rapid review findings.

Due to heterogeneity of the data from the included studies, a meta-analysis could not be conducted. Rather, the evidence was synthesized narratively and thematically according to the social dimensions and functions of the interventions and social factors considered. The analysis focused on the characteristics of social capital interventions and their impact on HPV immunization and cervical cancer screening (e.g., uptake, knowledge, intentions).

## Results

### Overview

**Figure 1:**
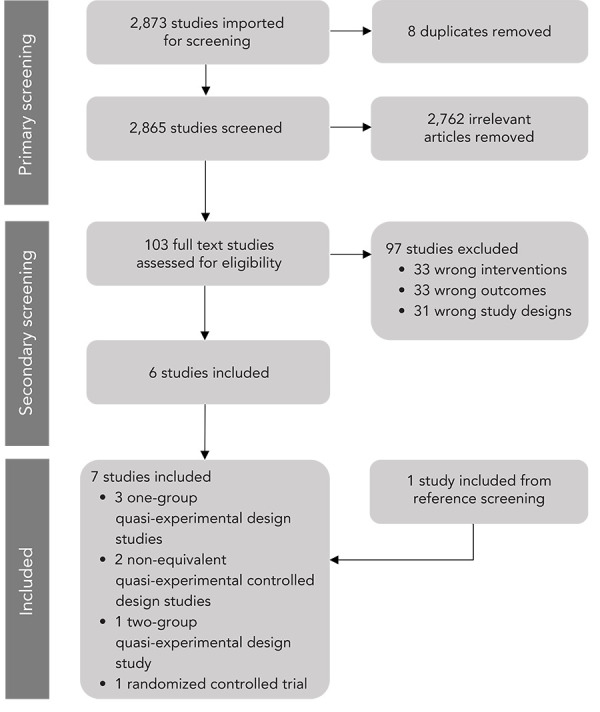
PRISMA chart of rapid review screening process

The search produced 2,873 studies. Through primary screening, 103 studies met the inclusion criteria. In the secondary screening, 97 studies were excluded. In the reference list screening process, one study met the inclusion criteria. This review included seven studies ((15–21)) (**Figure 1**).

### Key characteristics

**Table 1** summarizes the key characteristics of the included studies. Most studies were conducted in the United States ((15,17–20)). Six were quasi-experimental studies ((15–18,20,21)) and one was a randomized control trial ((19)). All seven studies had an educational component. Six studies incorporated culture into the educational component by utilizing co-ethnic health professionals or lay health educators who came from the same ethnic groups and/or spoke the same language as the participants ((15,17–21)). All seven studies included a cognitive dimension of social capital and two studies had a structural dimension of social capital ((19,21)). All studies had a bonding and bridging function of social capital and five had a linking component ((16,17,19–21)). Six studies had a “weak” quality rating score ((15,17–21)) and one received a “moderate” rating ((16)) (Appendix, Table A2). Overall, the evidence was weak due to data collection methods, withdrawal reporting and limitations of blinding.

**Table 1:** Description of main study characteristics

Characteristics	Categories	Number (n); proportion (%)	Reference
Location	United States	n=5; 71.4%	Chu *et al.*, 2021; Larkey *et al.*, 2012; Ma *et al.*, 2022; McDonough *et al.*, 2016; Lee *et al.*, 2018
	Iran	n=1; 14.3%	Khani Jeihooni *et al.*, 2021
	Nigeria	n=1; 14.3%	Olubodun *et al.*, 2022
Study design	One-group quasi-experimental study	n=3; 42.9%	Chu *et al.*, 2021; Ma *et al.*, 2022; McDonough *et al.*, 2016
	Non-equivalent quasi-experimental controlled study	n=2; 28.6%	Khani Jeihooni *et al.*, 2021; Olubodun *et al.*, 2022
	Two-group quasi-experimental study	n=1; 14.3%	Larkey *et al.*, 2012
	Randomized controlled trial (RCT)	n=1; 14.3%	Lee *et al.*, 2018
Interventions	Educational component	n=7; 100%	Chu *et al.*, 2021; Khani Jeihooni *et al.*, 2021; Larkey *et al.*, 2012; Lee *et al.*, 2018; Ma *et al.*, 2022; Olubodun *et al.*, 2022; McDonough *et al.*, 2016
	Co-ethnic/speaks the same language as participants’	n=6; 85.7%	Chu *et al.*, 2021; Larkey *et al.*, 2012; Ma *et al.*, 2022; Olubodun *et al.*, 2022; McDonough *et al.*, 2016; Lee *et al.*, 2018
HPV-related outcomes	Cervical cancer screening	n=5; 71.4%	Khani Jeihooni *et al.*, 2021; Larkey *et al.*, 2012; Ma *et al.*, 2022; Olubodun *et al.*, 2022; McDonough *et al.*, 2016
	HPV immunization	n=2; 28.6%	Chu *et al.*, 2021; Lee *et al.*, 2018
Social capital dimensions	Cognitive	n=7, 100%	Chu *et al.*, 2021; Khani Jeihooni *et al.*, 2021; Larkey *et al.*, 2012; Lee *et al.*, 2018; Ma *et al.*, 2022; Olubodun *et al.*, 2022; McDonough *et al.*, 2016
	Structural	n=2; 28.6%	Lee *et al.*, 2018; Olubodun *et al.*, 2022
Social capital functions	Bonding	n=7, 100%	Chu *et al.*, 2021; Khani Jeihooni *et al.*, 2021; Larkey *et al.*, 2012; Lee *et al.*, 2018; Ma *et al.*, 2022; Olubodun *et al.*, 2022; McDonough *et al.*, 2016
	Bridging	n=7, 100%	Chu *et al.*, 2021; Khani Jeihooni *et al.*, 2021; Larkey *et al.*, 2012; Lee *et al.*, 2018; Ma *et al.*, 2022; Olubodun *et al.*, 2022; McDonough *et al.*, 2016
	Linking	n=5; 71.4%	Khani Jeihooni *et al.*, 2021; Larkey *et al.*, 2012; Lee *et al.*, 2018; Olubodun *et al.*, 2022; McDonough *et al.*, 2016

Abbreviation: HPV, human papillomavirus

### Impact on human papillomavirus immunization

Only two studies reported the impact of social capital on HPV immunization ((15,19)) (**Table 2**). Factors associated with uptake included: HPV immunization-related knowledge; perceptions about one’s susceptibility to HPV; understanding the risks of HPV-related diseases and benefits of the immunization; intentions to be vaccinated for HPV; and immunization behaviours. One culturally appropriate, community-based education program delivered by co-ethnic health professionals resulted in significant improvement in mothers’ knowledge, beliefs and intentions to immunize their own children ((15)). However, there were no statistically significant differences in HPV immunization uptake among children within a six-month time frame. A narrative intervention also resulted in higher levels of intention to immunize among girls, but no differences in actual HPV immunization uptake ((19)). Due to the combination of multiple components (e.g., social capital and education) in the intervention, the effects of each component on the outcomes were not described. Despite improving knowledge, beliefs and intentions around HPV immunization, both studies reported the ineffectiveness of educational and narrative interventions in improving HPV immunization uptake in girls and their mothers (15,19).

**Table 2:** Characteristics of the social capital interventions and their impacts on human papillomavirus immunization

Study (in alphabetical order)	Objective	Country, population size and description	Description of intervention	Social capital dimensions	Social capital functions	Impact and effectiveness
Chu *et al.*, 2021	This one-group quasi-experimental study evaluated the impact of a culturally developed educational intervention for East African immigrant mothers to improve HPV vaccination knowledge, attitudes and intentions to vaccinate their male and female children.	United States 120 participants Sex: female, 100% Age: <30 years, 2.6%; 30–39 years, 57.0%; 40–49 years, 33.3%; ≥50 years, 7.0%	A socio-context framework and Andersen’s behavioural model were applied to include social, cultural and religious factors to inform a community-based education intervention delivered by co-ethnic health professionals. A communal dinner for all participating mothers and their children was held prior to the implementation of the education forum. The forum included a 40-minute interactive session with the co-ethnic health professional, a 20-minute presentation in the participants’ native languages and a 20-minute question and answer period.	**Cognitive:** · Social norms and influences were measured using survey items. · Focus group findings deepened the understanding of social influences (social, cultural, religious factors). These findings on contextual factors informed the development of the intervention.	**Bridging and bonding:** · The intervention was designed to be sensitive, language and culturally appropriate and audience-centric to appeal to the East African community.	· Within 6 months of the intervention, only 2% (n=2) of the 96 mothers with children who had no HPV vaccination records received the HPV vaccine. · The proportion of mothers who wanted to vaccinate their children increased after intervention (6.3%; n=7/111 to 75.7%; n=84/111). · Post-intervention, 86.4% (n=95/110) of mothers reported that they were more likely to talk with their children’s doctors about the HPV vaccine than pre-intervention (*p*<0.0001). · Post-intervention, mothers had a significant increase in knowledge and beliefs about HPV (*p*<0.0001; RR 3.64; 95% CI: 2.89–4.60), HPV vaccination (*p*<0.0001; RR 8.10; 95% CI: 5.26–12.45) and reported positive HPV vaccination intentions (*p*<0.0001; RR 5.03; 95% CI: 3.42–7.39). · Post-intervention, 90.2% (n=101/112) of mothers thought they had enough information to make a decision about vaccinating their children and 92.4% (n=97/105) knew where to get the HPV vaccination compared to baseline (11.6%; n=13 and 25.7%; n=27 respectively; *p*<0.0001).
Lee *et al.*, 2018	This randomized controlled trial examined the feasibility, acceptability and effectiveness of a narrative intervention to promote HPV immunization in Cambodian mothers and daughters.	United States 18 dyads (38 total mothers and daughters), 9 in the intervention and 9 in the control group. Mean age: daughters, 15.3 years old; mothers, 44.9 years old	The intervention included a storytelling narrative of HPV immunization, which was informed by the network episode model. This model describes that interpersonal interactions (e.g., peer influence) within social networks function as a mechanism for health-related decision-making; thus, it is both a social and individual process. The storytelling narrative was a 26-minute storytelling DVD that utilized unscripted, culturally grounded stories in the first person. The real stories increased realism by recruiting important people from the Khmer community, such as physicians and community members who were both vaccinated and unvaccinated. The control group received non-narrative education materials.	**Structural:** · Narrative intervention employed community members, friends, family and doctors (social networks) to encourage vaccination behaviours. **Cognitive:** · The storytelling narrative was developed by other Khmer mothers, daughters and community health leaders. · Participants were recruited through community health leaders, site coordinators and cultural navigators’ social networks in addition to other methods, such as advertising on local radios.	**Linking:** · Trusted community health leaders utilized their social networks to aid in study recruitment. **Bridging:** · Participants, community health leaders and actors within the storytelling narrative were all part of the Khmer community. While these groups share similar characteristics or identities, they are part of different networks. **Bonding:** · Dyads of mothers and daughters were recruited because mothers are the primary health decision-makers for their daughters.	· Within one month, daughters from the intervention group reported higher intentions to receive HPV immunization than their control group counterparts. However, there was no difference in actual vaccination initiation between both groups. · Storytellers shared how they were personally influenced by their social networks and norms from friends, mothers and healthcare providers to receive the HPV vaccination. · Social network norms were effective in motivating the vaccination intentions of participants through a positive emotional reaction. Note: No statistical data was provided.

Abbreviations: CI, confidence interval; HPV, human papillomavirus; RR, relative risk

### Impact on cervical cancer screening

Five studies found mixed results regarding the impact of social capital on cervical cancer screening (16–18,20,21) (**Table 3**). One study on Pap smear testing found no significant differences in subjective norms and perceived behavioural control between the groups receiving and not receiving an educational intervention ((16)). However, these factors increased significantly among the participants within the education intervention groups, according to pre-post analysis. Two other studies found that the group format of the educational sessions contributed to higher overall scores in emotional, instrumental, reciprocal and perceived social support ((17,18)). One study in local community and faith-based settings examined the knowledge, attitudes and uptake of HPV self-sampling tests that were provided by bilingual health educators ((18)). All participants completed the HPV self-sample test, with most participants reporting that they were “comfortable/very comfortable” with self-sampling.

**Table 3:** Characteristics of the social capital interventions and their impacts on cervical cancer screening

Study (in alphabetical order)	Objective	Country, population size and description	Description of intervention	Social capital dimensions	Social capital functions	Impact and effectiveness
Khani Jeihooni *et al.*, 2021	This non-equivalent quasi-experimental controlled study examined the effect of a Pap smear educational intervention targeting the beliefs, subjective norms and perceived behavioural control in Iranian women.	Iran 300 women (150 in the control group and 150 in the experimental group).	Health belief model and theory of planned behaviour were used to inform an educational program that was based on active learning to enhance the knowledge of cervical cancer, Pap smear tests, barriers to screening and individual and social factors related to Pap smear testing. The experimental group participated in eight 50-minute education sessions once per week that included a group discussion, brainstorming, question and answer and a film display to facilitate motivation and behavioural control in Pap smear testing. Spouses, physicians and healthcare staff were present during these sessions to play supporting roles. These groups helped to influence the subjective norms around cervical cancer screening. Control group participants received no education intervention.	**Cognitive:** · The health belief model, informing the educational intervention, depicts subjective norms as a result of many normative beliefs and perceptions; thus, people will often act based on their perception of what others would think they should do.	**Linking, bridging and bonding:** · The intervention included an educational session with spouses, physicians and health centre staff in attendance to play supporting roles and influence the subjective norms around screening behaviours.	· At 6-month post-intervention, a significantly greater portion of the experimental group received the Pap smear test (72%; n=108/150), compared to the control group (6%; n=9/150; *p*<0.05). · There was no significant difference in knowledge (*p*=0.09), perceived susceptibility to HPV and associated diseases (*p*=0.104) and severity of cervical cancer (*p*=0.135), barriers (*p*=0.121), benefits of cervical cancer screening (*p*=0.176), behavioural control (*p*=0.289), subjective norms (*p*=0.322), or intention scores (*p*=0.355) between control and experimental groups at baseline. · At 6-month post-intervention, there was a significant improvement in knowledge (*p*<0.05), understanding of perceived susceptibility to and severity of cervical cancer (*p*<0.05) and benefits of cervical cancer screening (*p*<0.05), behavioural control (*p*<0.05) and subjective norms (*p*<0.05) in the experimental group compared to the control group. Within the control group, there were no significant changes (*p*>0.05). · At 6-month post-intervention, there was a significant decrease in perceived barriers to cervical cancer screening (*p*<0.05), such as lack of time, in the experimental group. Within the control group, there were no significant changes (*p*>0.05).
Larkey *et al.*, 2012	This two-group quasi-experimental design study examined the effect of using lay health educators to increase cancer screening behaviours in Latinas.	United States 1,006 women (604 women in social support group [SSG] and 402 women in individual [IND] group). Age: mean of 38.4 years old	The same intervention was delivered in two different formats: IND and SSG. The intervention included six 80-minute educational sessions that contained definitions for different cancers; dietary, tobacco and physical activity recommendations for each cancer (cervical, breast and colorectal); and screening information. The SSG intervention was designed to promote group interactions and involvement to encourage women to meet each other’s needs and have group goal setting.	**Cognitive:** · A Hispanic Advisory Board reviewed the intervention educational curriculum. They provided insight into how to organize groups and develop a sense of identity and commitment within a group.	**Linking:** · Lay health educators were considered “practical supports”, as individuals who can share health information with others. **Bridging and bonding:** · Lay health educators (or *promotoras de salud*) were language-matched and networked in their communities.	· No significant differences in cervical cancer screening between the SSG and IND groups (*p*=0.315). · No significant differences in maintenance of cervical cancer screening (*p*=0.971).
Ma *et al.*, 2022	This one-group quasi-experimental design study evaluated the impact of a culturally tailored intervention for Chinese, Korean and Vietnamese women on HPV self-sampling test uptake.	United States 156 Asian-American women Age: mean of 44.66 years old	The intervention was informed by the health belief model and the community-based participatory research approach. The intervention contained four different components: group education workshops, written and illustrated instructions on the HPV self-sampling test, group discussion session and patient navigation and follow-up care.	**Cognitive:** · Focus groups informed the cultural components of the intervention. · Perceived social support was assessed using 11 survey questions to measure support from spouses, other family members, friends and physicians related to cervical cancer screening.	**Bridging and bonding:** · The intervention contained a group education component with bilingual health educators.	· 100% (n=156/156) of the participants completed the HPV self-sampling test, but only 92.5% (n=145/156) were adequate samples. · HPV-related knowledge, social support, self-efficacy and comfort increased significantly following the intervention (*p*<0.001).
McDonough *et al.*, 2016	This one-group quasi-experimental design study evaluated the effectiveness of an educational intervention to improve Latina’s knowledge, attitudes, behaviours and intentions to get the Pap smear test.	United States 5,211 Latina women Age: mean of 39.07 years old	The intervention included an educational curriculum toolkit for *promotores de salud* (community health workers) to use in delivering cervical cancer screening education to Spanish-speaking Latina women. The toolkit contained bilingual materials of flip charts, key talking points, a *charla* (health education session) guide, educational brochures and a list of local resources for low-cost or free Pap smear testing.	**Cognitive:** · *Promotores de salud* offered social support, a sense of belonging and trust.	**Linking:** · *Promotores de salud* lived in the communities and provided health services and education as trusted members of the community. They acted as cultural brokers between the communities and the healthcare system. **Bridging and bonding:** · The intervention was delivered to a group of participants that identified as Latina and were part of a culturally similar group.	· Intentions to receive a Pap smear test increased significantly (*z*=−8.94; *p*<0.001). · Knowledge (*p*<0.001; 95% CI: −2.67, −2.53; r=0.73), positive attitudes (*p*<0.001; 95% CI: −0.15, −0.12; r=0.29) and self-efficacy (*p*<0.001; 95% CI: −0.18, −0.15; r=0.29) related to cervical cancer prevention and screening increased significantly.
Olubodun *et al.*, 2022	This non-equivalent quasi-experimental controlled study examined the effects of a social marketing intervention on Pap smear knowledge, attitudes and behaviours among women living in urban slums.	Nigeria 400 women (200 in the intervention group and 200 in the control group). Age: 21–30 years, 44.1%; 31–40 years, 31.7%; 41–50 years, 18.1%; 51–60 years, 3.8%; 60–65 years, 2.2%	The intervention was informed by the health belief model and focus groups. The intervention group received six health education sessions on cervical cancer and Pap smears, which included education for participants’ husbands. As part of the social marketing intervention, community mobilization was implemented to recruit key community members such as religious clerics and community leaders to publicly show support for cervical cancer screening. The control group also received health education sessions on cervical cancer and free Pap smear tests following the study.	**Structural and cognitive:** · The development of the intervention was informed by perceived barriers related to religion, culture, spouses’ disapproval and feelings of embarrassment. · Religious leaders, traditional leaders and husbands helped promote the Pap smear services through speeches at health education sessions.	**Bridging and bonding:** · People were assigned to groups based on similar sociodemographic characteristics, beliefs, values and behaviours. · Sensitization and educational sessions were targeted toward husbands to reduce spouses’ disapproval.	· Cervical cancer screening uptake significantly increased in the intervention group (0% to 84.3%; *p*<0.001; 95% CI: 0.8–0.9), but not in the control group (*p*=1.000). · Change in knowledge was statistically significant in the intervention group (mean=0.0, SD=0.3 to mean=15.1, SD=3.7; *p*<0.001; 95% CI: 14.3–15.6), but not in the control group (*p*=0.096). · Attitude scores improved significantly in the intervention group (mean=27.2, SD=1.4 to mean=36.5, SD=4.8; *p*<0.001; 95% CI: 8.5–10.1), but not in the control group (*p*=0.068).

Abbreviations: CI, confidence interval; HPV, human papillomavirus; r, effect size; SD, standard deviation; *z*, *z* score

Groups receiving educational interventions reported outcomes that included increased knowledge related to cervical cancer and screening procedures, improved understanding of perceived susceptibility to HPV (i.e., the belief that one is likely to get HPV or HPV-related disease), severity of cervical cancer (i.e., risk and seriousness of HPV, HPV-related disease and associated complications to one’s life), benefits of cervical cancer screening (i.e., reduction of risk and severity of getting HPV and HPV-related disease), increased intentions for cervical cancer screening uptake and greater uptake of the Pap smear test (e.g., administered by a physician or HPV self-sampling test) (16,18,20,21). Among the four studies that included uptake measures (12–14,17), three reported increased cervical cancer screening uptake (16,18,21). One study found no significant differences in cervical cancer screening uptake between the cohort receiving education sessions in groups to promote social capital and the cohort receiving the session individually with no social capital component (17). However, it also found that cervical cancer screening increased in both group and individual education sessions.

### Equity considerations

**Table 4** presents equity-related findings on HPV immunization and cervical cancer. The studies either tailored their interventions to meet the needs of specific groups or described results for specific disadvantaged groups (e.g., immigrants) considering, for example, education level and gender and/or sex.

**Table 4:** Summary of equity considerations in the included studies

Social factors according to PROGRESS-Plus	Findings
Education, place of residence and socioeconomic status	· Knowledge, attitudes, intentions and behaviours related to HPV immunization and cervical cancer screening were improved by creating an enabling environment in low-income countries facing poor access to health services, long hospital wait times, lower education levels, lack of basic amenities (e.g., latrines and safe running water) and higher prevalence of risky sexual behaviours (Khani Jeihooni *et al.*, 2021; Olubodun *et al.*, 2022). · The majority of population groups studied received a high school education or less, which had implications on how the educational components of the intervention were designed (e.g., delivered verbally through lay health advisors, promoters, mixed marketing approach, PowerPoint) (Chu *et al.*, 2021; Khani Jeihooni *et al.*, 2021; Larkey *et al.*, 2012; Lee *et al.*, 2018; Ma *et al.*, 2022; McDonough *et al.*, 2016; Olubodun *et al.*, 2022). · Given the majority of the population groups were from low-income households or lived in poverty (Chu *et al.*, 2021; Khani Jeihooni *et al.*, 2021; Larkey *et al.*, 2012; Ma *et al.*, 2022; McDonough *et al.*, 2016; Olubodun *et al.*, 2022), provision of free Pap tests or referrals reduced cost barriers (especially for those who were uninsured) to receiving cervical cancer screening (McDonough *et al.*, 2016; Olubodun *et al.*, 2022).
Language	· Given language negatively affected knowledge and confidence in HPV-related decision-making, interventions provided multiple translated versions of their materials for their target population (Chu *et al.*, 2021; Larkey *et al.*, 2012; Lee *et al.*, 2018; Ma *et al.*, 2022; McDonough *et al.*, 2016; Olubodun *et al.*, 2022). · Participants preferred community classes delivered in the community’s native language, which facilitated community dialogue and reduced mistrust of immunization and healthcare (Chu *et al.*, 2021).
Race, ethnicity, religion and culture	· Racial and ethnic minority groups in the United States have lower uptake of HPV immunization and cervical cancer screening due to limited awareness and lack of knowledge; language barriers; physical barriers (e.g., transportation and time to get to clinics); misperceptions about efficacy and safety regarding HPV immunization; mistrust of healthcare or immunization; lack of strong healthcare provider recommendations; healthcare costs (e.g., lack of insurance); and cultural beliefs, norms (e.g., restrictions around pork products) and stigma (e.g., association between getting the HPV vaccine and increasing sexual behaviours) (Chu *et al.*, 2021; Larkey *et al.*, 2012; Ma *et al.*, 2022). · Culturally appropriate interventions resulted in significant improvement in mothers’ confidence, knowledge, beliefs and intentions to immunize their own children (Chu *et al.*, 2021). · Several studies utilized focus groups, stakeholder feedback and consultations with community leaders to inform their research design to create culturally relevant, community-based and audience-sensitive and specific content (Chu *et al.*, 2021; Larkey *et al.*, 2012; Lee *et al.*, 2018; Ma *et al.*, 2022; McDonough *et al.*, 2016). · Inviting community members and organizations to support HPV immunization initiatives (e.g., sharing the HPV immunization program with their communities) had a positive effect on participant recruitment among racial and ethnic groups (Chu *et al.*, 2021; Ma *et al.*, 2022). · Storytelling narratives effectively increased HPV immunization intentions (Lee *et al.*, 2018). · Delivery of an immunization information by co-ethnic research assistants was found to be successful in promoting behaviour changes in target populations (Chu *et al.*, 2021). · Trusted community members (e.g., lay health advisors, patient navigators) were found to have the ability to broker the relationships between healthcare providers and target population groups and act on their established social networks to diffuse information into the communities (Larkey *et al.*, 2012; McDonough *et al.*, 2016).
Gender and/or sex	· HPV immunization target populations were predominantly specified as girls and women (Chu *et al.*, 2021; Khani Jeihooni *et al.*, 2021; Larkey *et al.*, 2012; Lee *et al.*, 2018; Ma *et al.*, 2022; McDonough *et al.*, 2016; Olubodun *et al.*, 2022). · Barriers for women to seek a Pap test included the painful nature of the test; shame attributed to getting tested; inadequate knowledge; cultural and religious beliefs; and psychosocial causes (e.g., subjective norms, social pressures, embarrassment) (Khani Jeihooni *et al.*, 2021). · Women who had adequate knowledge of cervical cancer were more likely to recognize the risks, severity, susceptibility and benefits of cervical cancer screening (Khani Jeihooni *et al.*, 2021). · Subjective norms, such as support of family members and healthcare staff cooperation, impacted the intention and behaviour of women to seek cervical cancer screening (Khani Jeihooni *et al.*, 2021). · Findings were mixed regarding the influence of fathers and husbands on women receiving cervical cancer screening and children’s decisions to receive HPV Immunization. One study indicated that Somali fathers had less influence than mothers on their decisions to immunize their children (Chu *et al.*, 2021). In some countries, husbands may need to consent before women are able to undergo cervical cancer screening. Thus, providing education sessions for husbands was recommended to reduce disapproval of screening (Olubodun *et al.*, 2022). · Overall, the reported preference to have a female sample collector for cervical cancer screening may indicate an opportunity to engage female physicians and nurses while reducing patients’ shyness and shame (Olubodun *et al.*, 2022).

## Discussion

To our knowledge, this is the first review of social capital interventions in public health regarding HPV immunization and cervical cancer screening. Despite interest in the use of social capital to improve cancer outcomes ((8,22,23)), only seven papers met this review’s inclusion criteria. Concerning primary prevention, education interventions containing social capital dimensions and/or functions were found to increase HPV immunization knowledge, attitudes and intentions. They successfully addressed concerns, fears and doubts for providing accurate information, building a trustworthy relationship between participants and researchers or providers and meeting participants’ life circumstances and sociocultural needs. However, they seemed to have failed in bridging the intention-uptake gap in HPV immunization. This finding speaks to the recognition that knowledge is only one of the multiple determinants of vaccine decision-making, as some vaccine-hesitant people delay or refuse vaccination after educational interventions ((24)). Pairing social capital interventions with a vaccine offer or immunization appointment scheduling at the end of the intervention may effectively increase uptake. For those with limited access to the healthcare system, school-based health outreach and partnerships with communities should be part of the strategy to build multisectoral delivery platforms for vaccination and to promote uptake following educational intervention ((25)).

Regarding secondary prevention, this review found that interventions improved several outcomes including knowledge on cervical cancer and screening procedures; understanding of perceived susceptibility to and severity of HPV infection and cervical cancer; benefits and intentions of cervical cancer screening; and emotional, instrumental, reciprocal and perceived social support. Among the four studies analyzing the uptake of cervical cancer screening, three found increased uptake. These three studies used the health belief model in the design of their interventions, which seeks to change an individual’s beliefs, knowledge and perceived benefits and risks to positively influence their health behaviours ((26)). This finding may indicate the value of using a theoretical health behaviour change model alongside dimensions of social capital to guide cervical cancer screening interventions. While our findings do not allow us to infer how much contribution social capital made on cervical cancer screening uptake, they indicate that social capital plays a role and should be a component in screening interventions. Further research should consider the influences of other factors on participation in cervical cancer screening (e.g., limited access to sexual and reproductive healthcare programs).

Consistent with the current literature, this review’s findings support the need for interventions to consider perceptions of social capital in different contexts and to reflect the multidimensional factors influencing people’s decision-making on HPV immunization and cervical cancer screening ((27)). To create an environment conducive to positive HPV-related knowledge, intentions and behaviours, social capital interventions should address perceived social and structural barriers like affordability and accessibility of immunization and screening programs. Anticipating contextual barriers that jeopardize the success of social capital interventions for increasing uptake requires moving away from half measures such as charging for HPV vaccines or limiting vaccination appointments to work hours. The World Health Organization has called for actions to ensure affordability and expansion of HPV vaccination and cervical cancer screening coverage ((28)), including single dose for adolescents to reduce costs and burden to the healthcare system and incorporation of cervical cancer screening into state health insurance schemes to address social inequities in secondary prevention. The World Health Organization also recommends developing partnerships between the public health sector and public, private and non-profit organizations to roll out services and address constraints in HPV vaccine supply and devices for cervical cancer diagnostics ((25,28)).

Most studies in this review specified their HPV immunization target populations as “girls” and “women” and only one included mention of “boys.” None of the studies focused on members of the Lesbian, Gay, Bisexual, Transgender, Queer and Questioning and Two-Spirit (LGBTQ2S+) community. This reflects an overlook of gender identity and sexual diversity in interventions utilizing social capital. Trends examining HPV immunization rates indicate a greater gap in HPV immunization rates among males generally and that HPV-related cancer rates are predicted to rise among populations who do not have a cervix ((29). This may be due to the prior focus of HPV vaccine promotions to prevent cervical cancer, which continues to act as a barrier for uptake of the newer nonavalent HPV vaccine that protects against oropharyngeal, anogenital and cervical cancer-causing strains of HPV. The LGBTQ2S+ community is more likely to experience an HPV infection and less likely to receive an HPV vaccine than heterosexual groups ((30–32)). Social support may support HPV vaccine uptake among LGBTQ2S+ people (33). As HPV infects both biological males and females and can lead to cancer in any person irrespective of their gender identity or sexual orientation, future research should expand the evidence base concerning interventions utilizing social capital targeting LGBTQ2S+ populations and biological males.

## Limitations

The strengths of this rapid review include the use of a systematic methodology for screening and data extraction and analysis, assessment of methodological quality and consideration of social factors. However, data synthesis was limited to a small sample of studies, which may reflect the heterogeneity of study designs and measures. As the included studies focused on interventions across the world, the generalizability, transferability and applicability of the review findings are context-dependent and the unique circumstances of each region and population should be considered. This creates opportunity for future research and implementation work focusing on the unique knowledge and awareness needs of each population, such that HPV immunization and cervical cancer screening is promoted as an autonomous, yet supported, culturally appropriate decision among disadvantaged populations.

## Conclusion

This rapid review examined the evidence concerning the characteristics and impact of interventions utilizing social capital on HPV immunization and cervical cancer screening. It found limited and mixed results regarding the use of social capital as a mechanism to improve uptake of HPV immunization and cervical cancer screening. However, evidence suggests that interventions that consider and reflect the local context may increase the uptake of HPV immunization and cervical cancer screening. Given the strength of evidence from experiments and quasi-experiments, more research using those design studies are needed to understand the impacts of social capital interventions on HPV immunization and cervical cancer screening. Health researchers examining those programs should consider designing interventions that include social capital components that, for instance, enhance participants’ trust of health practitioners and engage with religious leaders. Public health agencies should consider the promising results of culturally appropriate and tailored interventions containing components of social capital for creating positive change in HPV-related knowledge, attitudes, intentions and behaviours toward HPV immunization and cervical cancer screening. Further research must translate these psychological changes into HPV immunization and cervical cancer screening behaviours.

## Appendix

**Table A1:** Inclusion and exclusion criteria

Characteristics	Inclusion criteria	Exclusion criteria
Population	No limitation on population. All populations included. Populations can include, but are not limited to: · School aged, HPV immunization-eligible children. · Adults eligible for HPV immunization (18–26 years of age). · Women and people with a cervix eligible for cervical cancer screening. · Adults at risk for HPV-associated cancers (i.e., head, neck, anal, vaginal, vulvar, penile, oropharynx cancer), including high-risk populations (e.g., men who have sex with men).	None.
Intervention	Policy/program interventions related to social capital (primordial intervention); as a mechanism to, or in combination with interventions that improve HPV immunization AND/OR cervical cancer screening. · Interventions should be group or community-based. · Interventions aimed at increasing social capital should be at the upstream or midstream levels. · Interventions that aim to build trust in the healthcare system or build rapport within the population group (e.g., HPV immunization education program that increases social capital). · Knowledge and attitude interventions designed in a culturally relevant way to promote bonding within family, reliance on others, sense of community and trust (e.g., community-based programs creating opportunities for social interactions among participants). Interventions that do not explicitly outline that it is aimed at increasing or contain components of social capital (e.g., structural or cognitive social capital), BUT reports on social capital outcomes are included. · Social media as a platform for intervention (e.g., online interventions) is included if it specifies that it aims to increase social capital (e.g., trust, rapport, peer support, family support, online relationships or connections) OR it reports social capital outcomes. · Intervention focusing on improving knowledge and attitudes should have outcomes related to social capital (e.g., peer support, perceived social norms from family).	Interventions that ONLY focus on staff training or education, coping strategies related to needle phobias/medical procedures, traumas, anxiety, etc. delivered by professionals. · Interventions that are targeted to be delivered one-on-one or at individual-level. · Interventions that aim to change behaviours. General immunizations not related to HPV. Screening for cancers that are not cervical cancer. Interventions that do not explicitly outline that it aims to increase social capital or contains components of social capital AND does not report on social capital outcomes are excluded.
Comparator	None or any, as relevant.	None.
Outcomes	Impact/effectiveness outcomes MUST be related to HPV immunization AND/OR cervical cancer screening (e.g., HPV immunization uptake or participation, cervical cancer screening initiation in never screeners, incidence of HPV-associated cancers or outcomes, HPV vaccine acceptance, HPV immunization or cervical cancer screening intentions). Interventions that do not explicitly state that they aim to increase social capital must report on social capital outcomes to be included.	Studies that do not report on outcomes associated with HPV immunizations, cervical cancer screening, or HPV-associated cancers. Studies that do not measure or evaluate the impact or effectiveness of HPV immunization, cervical cancer screening, or HPV-associated cancers. Studies that only report changes in social capital or health inequities.
Setting	No limitation on settings. This includes, but is not limited to, healthcare settings, community-based settings and school-based settings. No limitation on geographical location. This includes, but is not limited to: · Urban locations · Rural locations · Suburban locations · Any country in the world	None.
Study design	Study is published in a peer-reviewed journal. Intervention studies: single group (pre-post), quasi-experimental (non-randomized interventions) and randomized controlled trials. Systematic reviews and meta-analyses that include any type of intervention studies as outlined above.	Primary research studies using qualitative methods and analysis. Observational studies, such as cohort, cross-sectional and case-control studies. Cost-effectiveness studies. Any other types of review, such as scoping reviews and narrative reviews. Descriptive studies and studies in the form of comments, editorials, letters to the editor, theoretical papers, books, book chapters, protocols, case studies, case reports, grey literature (e.g., magazine articles, dissertations, doctoral theses, conference papers, position statements, preprints). Systematic reviews and meta-analyses, including intervention studies (in inclusion criteria) and other types of study designs (outlined in exclusion criteria above), will be excluded, unless findings are reported separately for intervention studies.
Language	Full text is published in English.	Only abstract in English.
Date	Publication date between 2012 and 2022 (last 10 years).	Publication date prior to 2012.

**Table A2:** Quality of quantitative studies reviewed using the Effective Public Healthcare Panacea Project (EPHPP) quality assessment tool (n=7)

Study (in alphabetical order)	Selection bias	Study design	Confounding	Blinding	Data collection method	Withdrawal	Final rating
Khani Jeihooni *et al.*, 2021	Moderate	Strong	Strong	Moderate	Strong	Weak	Moderate
Chu *et al.*, 2021	Moderate	Moderate	Strong	Weak	Weak	Strong	Weak
Larkey *et al.*, 2012	Moderate	Strong	Strong	Moderate	Weak	Weak	Weak
Lee *et al.*, 2018	Weak	Strong	Weak	Moderate	Weak	Strong	Weak
Ma *et al.*, 2022	Weak	Moderate	Strong	Weak	Weak	Strong	Weak
McDonough *et al.*, 2016	Moderate	Moderate	Strong	Weak	Moderate	Weak	Weak
Olubodun *et al.*, 2022	Moderate	Strong	Weak	Moderate	Weak	Moderate	Weak

## References

[1] Government of Canada. Human papillomavirus vaccine: Canadian immunization guide. Ottawa, ON: Government of Canada; 2021. [Accessed 2023 June 8]. https://www.canada.ca/en/public-health/services/publications/healthy-living/canadian-immunization-guide-part-4-active-vaccines/page-9-human-papillomavirus-vaccine.html

[2] Bruni L, Albero G, Serrano B, Mena M, Collado JJ, Gómez D. Human papillomavirus and related diseases report. 2023. https://hpvcentre.net/statistics/reports/CAN.pdf?t=1565188933974

[3] Volesky KD, El-Zein M, Franco EL, Brenner DR, Friedenreich CM, Ruan Y; ComPARe Study Team. Cancers attributable to infections in Canada. Prev Med 2019;122:109–17. 10.1016/j.ypmed.2019.03.035 31078164

[4] Caird H, Simkin J, Smith L, Van Niekerk D, Ogilvie G. The path to eliminating cervical cancer in Canada: Past, present and future directions. Curr Oncol 2022;29(2):1117–22. 10.3390/curroncol29020095 35200594 PMC8870792

[5] Canadian Partnership Against Cancer. Action plan for the elimination of cervical cancer in Canada, 2020–2030. 2020. [Accessed 2023 June 8]. https://www.partnershipagainstcancer.ca/topics/elimination-cervical-cancer-action-plan/

[6] Canadian Partnership Against Cancer. HPV vaccine access in Canada, 2022. 2022. [Accessed 2024 Mar 27]. https://www.partnershipagainstcancer.ca/topics/hpv-vaccine-access-2022/

[7] Canadian Partnership Against Cancer. HPV immunization for the prevention of cervical cancer. 2021. [Accessed 2024 Mar 27]. https://www.partnershipagainstcancer.ca/topics/hpv-immunization-policies/

[8] Moudatsou MM, Kritsotakis G, Alegakis AK, Koutis A, Philalithis AE. Social capital and adherence to cervical and breast cancer screening guidelines: a cross-sectional study in rural Crete. Health Soc Care Community 2014;22(4):395–404. 10.1111/hsc.12096 24450830

[9] Moore S, Kawachi I. Twenty years of social capital and health research: a glossary. J Epidemiol Community Health 2017;71(5):513–7. 10.1136/jech-2016-208313 28087811

[10] Tricco AC, Langlois EV, Straus SE. Rapid reviews to strengthen health policy and systems: A practical guide. Geneva, CH: WHO; 2017. http://apps.who.int/iris/bitstream/10665/258698/1/9789241512763-eng.pdf 10.1136/bmjgh-2018-001178 PMC6407563 30899562

[11] Dobbins M. Rapid review guidebook: Steps for conducting a rapid review. The National Collaborating Centre for Methods and Tools (NCCMT); 2017. https://www.nccmt.ca/uploads/media/media/0001/01/a816af720e4d587e13da6bb307df8c907a5dff9a.pdf

[12] Cochrane Methods Equity. PROGRESS-Plus. 2023. [Accessed 2022 June 22]. https://methods.cochrane.org/equity/projects/evidence-equity/progress-plus

[13] Effective Public Healthcare Panacea Project. Quality assessment tool for quantitative studies. 2023. [Accessed 2022 Dec 7]. https://www.ephpp.ca/quality-assessment-tool-for-quantitative-studies

[14] Page MJ, McKenzie JE, Bossuyt PM, Boutron I, Hoffmann TC, Mulrow CD, Shamseer L, Tetzlaff JM, Akl EA, Brennan SE, Chou R, Glanville J, Grimshaw JM, Hróbjartsson A, Lalu MM, Li T, Loder EW, Mayo-Wilson E, McDonald S, McGuinness LA, Stewart LA, Thomas J, Tricco AC, Welch VA, Whiting P, Moher D. The PRISMA 2020 statement: an updated guideline for reporting systematic reviews. BMJ 2021;372(71):n71. 10.1136/bmj.n71 33782057 PMC8005924

[15] Chu H, Ko LK, Ibrahim A, Bille Mohamed F, Lin J, Shankar M, Amsalu F, Ali AA, Richardson BA, Taylor VM, Winer RL. The impact of an educational forum intervention on East African mothers’ HPV vaccine-related knowledge, attitudes, and intentions to vaccinate their adolescent children. Vaccine 2021;39(28):3767–76. 10.1016/j.vaccine.2021.05.029 34053792 PMC9984200

[16] Khani Jeihooni A, Jormand H, Harsini PA. The effect of educational program based on beliefs, subjective norms and perceived behavior control on doing pap-smear test in sample of Iranian women. BMC Womens Health 2021;21(1):290. 10.1186/s12905-021-01419-w 34362375 PMC8348997

[17] Larkey LK, Herman PM, Roe DJ, Garcia F, Lopez AM, Gonzalez J, Perera PN, Saboda K. A cancer screening intervention for underserved Latina women by lay educators. J Womens Health (Larchmt) 2012;21(5):557–66. 10.1089/jwh.2011.3087 22416791

[18] Ma GX, Zhu L, Zhai S, Lin TR, Tan Y, Johnson C, Fang CY, Belinson JL, Wang MQ. Empowering low-income Asian American women to conduct human papillomavirus self-sampling test: A community-engaged and culturally tailored intervention. Cancer Control 2022;29:10732748221076813. 10.1177/10732748221076813 35193408 PMC8874186

[19] Lee H, Kim M, Cooley ME, Kiang PN, Kim D, Tang S, Shi L, Thiem L, Kan P, Peou S, Touch C, Chea P, Allison J. Using narrative intervention for HPV vaccine behavior change among Khmer mothers and daughters: A pilot RCT to examine feasibility, acceptability, and preliminary effectiveness. Appl Nurs Res 2018;40:51–60. 10.1016/j.apnr.2017.12.008 29579499

[20] McDonough AM, Vargas M, Nguyen-Rodriguez S, Garcia M, Galvez G, Rios-Ellis B. Mujer Sana, Familia Fuerte: the effects of a culturally-relevant, community-based, promotores program to increase cervical cancer screening among Latinas. J Health Care Poor Underserved 2016;27(2):568–79. 10.1353/hpu.2016.0094 27180696 PMC5929987

[21] Olubodun T, Balogun MR, Odeyemi KA, Osibogun A, Odukoya OO, Banjo AA, Sonusi SE, Olubodun AB, Ogundele OO, Dolapo DC. Effect of social marketing on the knowledge, attitude, and uptake of pap smear among women residing in an urban slum in Lagos, Nigeria. BMC Womens Health 2022;22(1):42. 10.1186/s12905-022-01620-5 35164717 PMC8842961

[22] Leader AE, Michael YL. The association between neighborhood social capital and cancer screening. Am J Health Behav 2013;37(5):683–92. 10.5993/AJHB.37.5.12 23985291

[23] Shelton RC, Gage-Bouchard EA, Jandorf L, Sriphanlop P, Thelemaque LD, Erwin DO. Examining social capital and its relation to breast and cervical cancer screening among underserved Latinas in the U.S. J Health Care Poor Underserved 2016;27(4):1794–811. 10.1353/hpu.2016.0163 27818439

[24] Dubé E, Gagnon D, Ouakki M, Bettinger JA, Guay M, Halperin S, Wilson K, Graham J, Witteman HO, MacDonald S, Fisher W, Monnais L, Tran D, Gagneur A, Guichon J, Saini V, Heffernan JM, Meyer S, Driedger SM, Greenberg J, MacDougall H; Canadian Immunization Research Network. Understanding vaccine hesitancy in Canada: Results of a consultation study by the Canadian Immunization Research Network. PLoS One 2016;11(6):e0156118. 10.1371/journal.pone.0156118 27257809 PMC4892544

[25] World Health Organization. Global strategy to accelerate the elimination of cervical cancer as a public health problem. Geneva, CH: WHO; 2020. https://www.who.int/publications/i/item/9789240014107

[26] Champion VL, Skinner CS. The health belief model. In: Glanz K, Rimer BK, Viswanath K, editors. Health behavior and health education: Theory, research, and practice. Jossey-Bass 2008. p. 45-65.

[27] Shiell A, Hawe P, Kavanagh S. Evidence suggests a need to rethink social capital and social capital interventions. Soc Sci Med 2020;257:111930. 10.1016/j.socscimed.2018.09.006 30219489

[28] World Health Organization. WHO cervical cancer eliminiation initiative: From call to action to global movement. Geneva, CH: WHO; 2023. [Accessed 2023 June 8]. https://www.who.int/publications/m/item/who-cervical-cancer-elimination-initiative--from-call-to-action-to-global-movement

[29] Diamond LM, Clarfield LE, Forte M. Vaccinations against human papillomavirus missed because of COVID-19 may lead to a rise in preventable cervical cancer. CMAJ 2021;193(37):E1467. 10.1503/cmaj.80082 34544788 PMC8476220

[30] Chidobem I, Tian F, Ogbuokiri E, Mgbodile F, Mgbodile C, Jokar TO, Shah MA, Pierre-Louis F. Trends in HPV and HPV vaccine awareness among gay and bisexual males in the U.S. Vaccines (Basel) 2022;10(4):604. 10.3390/vaccines10040604 35455355 PMC9032332

[31] Singh V, Gratzer B, Gorbach PM, Crosby RA, Panicker G, Steinau M, Amiling R, Unger ER, Markowitz LE, Meites E. Transgender women have higher human papillomavirus prevalence than men who have sex with men-two U.S. cities, 2012-2014. Sex Transm Dis 2019;46(10):657–62. 10.1097/OLQ.0000000000001051 31517805 PMC6849503

[32] Reiter PL, Bustamante G, McRee AL. HPV vaccine coverage and acceptability among a national sample of sexual minority women ages 18-45. Vaccine 2020;38(32):4956–63. 10.1016/j.vaccine.2020.06.001 32536546 PMC7323872

[33] Hao Z, Guo Y, Bowling J, Ledenyi M. Facilitators and barriers of HPV vaccine acceptance, initiation, and completion among LGBTQ community in the U.S.: A systematic review. Int J Sex Health 2021;34(2):291–307. 10.1080/19317611.2021.1989535 38596525 PMC10903696

